# Increasing Life Expectancy in Patients with Genitourinary Malignancies: Impact of Treatment Burden on Disease Management and Quality of Life

**DOI:** 10.1016/j.eururo.2024.11.026

**Published:** 2024-12-19

**Authors:** Marie-Pier St-Laurent, Bernard Bochner, James Catto, Benjamin J. Davies, Christian Daniel Fankhauser, Tullika Garg, Jill Hamilton-Reeves, Viraj Master, Bente T. Jensen, Susanne V. Lauridsen, Elizabeth Wulff-Burchfield, Sarah P. Psutka

**Affiliations:** a Department of Urologic Sciences, University of British Columbia, Vancouver, BC, Canada; b Urology Service, Department of Surgery, Memorial Sloan Kettering Cancer Center, New York, NY, USA; c Department of Urology, Sheffield Teaching Hospitals NHS Foundation Trust, Sheffield, UK; d Department of Urology Division of Health Services Research University of Pittsburgh School of Medicine, University of Pittsburgh Medical Center, Pittsburgh, PA, USA; e University of Lucerne, Lucerne, Switzerland; f University of Zurich, Zurich, Switzerland; g Department of Urology, Penn State Health Milton S. Hershey Medical Center, Hershey, PA, USA; h Department of Urology, University of Kansas Medical Center, Kansas City, KS, USA; i Department of Urology, Emory University School of Medicine, Atlanta, GA, USA; j Department of Urology, Aarhus University Hospital, Aarhus, Denmark; k WHO-CC/Clinical Health Promotion Centre, the Parker Institute, Bispebjerg and Frederiksberg Hospital, Frederiksberg, Denmark; l Center for Perioperative Optimization, Department of Surgery, Copenhagen University Hospital - Herlev and Gentofte, Herlev, Denmark; m Department of Clinical Medicine, University of Copenhagen, Copenhagen, Denmark; n Medical Oncology Division and Palliative Medicine Division, Department of Internal Medicine, University of Kansas School of Medicine, University of Kansas Cancer Center, The University of Kansas Health System, Kansas City, KS, USA; o Department of Urology, University of Washington, Seattle, WA, USA; p Clinical Research Division, Fred Hutchinson Cancer Center, Seattle, WA, USA

**Keywords:** Clinical trials, Genitourinary malignancies, Health-related quality of life, Overall survival, Patient-reported outcomes, Treatment burden

## Abstract

**Background and objective:**

Treatment burden refers to the overall impact of medical treatments on a patient’s well-being and daily life. Our objective is to evaluate the impact of treatment burden on quality of life (QoL) in patients with genitourinary (GU) malignancies, highlighting the importance of patient-reported outcomes (PROs) in clinical trials to inform treatment decisions and improve patient care.

**Methods:**

We conducted a narrative review of clinical trials focused on GU malignancy (prostate, bladder, and kidney) between January 2000 and June 2024, analyzing related PROs and findings regarding treatment burden.

**Key findings and limitations:**

Recent landmark clinical trials demonstrate significant improvements in overall survival across GU malignancies with novel therapies. However, the reporting of QoL outcomes in these trials is often inadequate, with many lacking comprehensive data or long-term impact. Current publications are increasingly evaluating treatment burden and its impact on patient well-being as a critical outcome, but most clinical trials to date have failed to assess treatment burden across key domains including financial, time and travel, and medication management.

**Conclusions and clinical implications:**

While advancements in treatment have extended longevity in patients with GU malignancies, the treatment burden associated with the receipt of novel agents and its implications for QoL remain inadequately uncharacterized.

## 1. Introduction

The global cancer burden is expected to reach 28.4 million cases by 2040, a 47% rise from 2020, with a larger increase in developing countries (from 64% to 95%) compared with developed countries (from 32% to 56%) [1]. Genitourinary (GU) malignancies (eg, prostate, bladder, and kidney) are significant contributors to this global burden, and the incidence is rising in tandem with a globally aging population and increasingly prevalent lifestyle-associated oncogenic risk factors such as obesity and smoking [2–4]. Age-standardized incidence rates (ASIRs) of bladder, prostate, and kidney cancers increased by 4%, 22%, and 29.1%, respectively, from 1990 to 2019 [3]. According to the Global Burden of Disease Study 2019, cancer remains a substantial and growing health challenge, with disparities in burden associated with the sociodemographic index [2].

As the prevalence of cancer continues to rise, it becomes increasingly crucial to address not only survival rates, but also the quality of life (QoL) of those affected during and after treatment. The World Health Organization defines QoL as “an individual’s perception of their position in life in the context of the culture and value systems in which they live and in relation to their goals, expectations, standards and concerns” [5]. Health-related quality of life (HrQoL) is a subset of QoL that specifically focuses on the impact of health status on these aspects of life, assessing how an individual’s physical (PWB), mental, emotional (EWB), and social (SWB) well-being are affected by a disease, disability, or treatment [6].

*Treatment burden* is a critical determinant of QoL, referring to the workload of receiving care and its impact on patients’ functioning and well-being [7,8]. Treatment burden is an emergent multidimensional concept incorporating self-management tasks that patients with chronic conditions must perform to manage a disease or medical condition, including medication adherence, dietary recommendations, and self-monitoring, among others [9]. It also encompasses the resources patients must mobilize to accomplish their health care work, including time, social support, and out-of-pocket costs [10]. Crucially, treatment burden is tied to the demands of managing the disease, not its natural progression [11].

Advancements in cancer treatment have led to improved survival rates for patients with GU malignancies [12]. However, despite these advances, there is a lack of comprehensive understanding of the cumulative treatment burden associated with newer therapies, and how this burden affects disease management and patient QoL. Informed decision-making requires that patients understand the potential impacts of their treatment choices not just on survival, but also on their overall well-being and daily functioning. They need accurate estimates of the potential tradeoffs regarding both QoL and treatment burden that they may face with different treatment approaches, to ensure that care decisions are in alignment with their individual priorities. This issue is becoming increasingly important as the global incidence of GU cancers continues to rise, and we have increasing access to novel therapies that increase longevity, leading to more patients undergoing prolonged and potentially intensive treatments with longer post-treatment survival.

Thus, our objective is to review the implications of novel therapeutics with respect to changes in life expectancy in the most common GU malignancies (prostate, bladder, and kidney) and to summarize the available evidence regarding the attendant impacts of these agents on QoL and treatment burden.

## 2. Methods

We conducted a narrative review evaluating changes in overall survival (OS) and QoL in patients with GU cancers, with a focus on the impact of contemporary and emerging treatments approved by major regulatory agencies and recommended in key clinical guidelines. We focused on prostate, bladder, and kidney cancers due to their high prevalence, significant impact of both diseases and relevant treatments on QoL, and ever increasing range of treatment modalities between which patients and clinicians must choose.

We conducted a comprehensive review of treatments approved for these GU malignancies by the FDA or European Medicines Agency, or recommended by the European Association of Urology, American Urological Association, or National Comprehensive Cancer Network guidelines. We selected treatments approved for both localized and advanced disease, reviewing phase 2 and 3 trials that led to their approvals through a guideline reference review as well as a review of key landmark trials identified by the panel of GU expert coauthors. We excluded studies not investigating prostate, bladder, or kidney cancer, or phase 1 studies given space constraints. We extracted data on OS and QoL from the identified studies, focusing on validated patient-reported outcome (PRO) instruments. For OS, key metrics such as hazard ratios (HRs), median survival times, survival rates, and *p* values were recorded. When multiple trials reported on similar disease stages, treatments, and/or pharmacological pathway, changes in OS were cited from the primary trial report with the most mature follow-up. Notably, our primary objective was not to critically appraise randomized controlled trial (RCT) quality or compare therapeutic approaches, but rather to summarize changing life expectancy across the primary GU malignancies with recent therapeutic advances. Second, for QoL assessment, the initial landmark trial identified previously was reviewed, and we summarized the reported QoL from validated PRO measures (PROMs). If no validated PROMs were reported, we performed target searches in PubMed and Embase of phase 2 or 3 trials that studied the same investigational treatment and used validated PROMs such as Functional Assessment of Cancer Therapy (FACT)–Bladder, FACT–Prostate, European Organisation for Research and Treatment of Cancer (EORTC) Quality of Life questionnaire (EORTC QLQ-C30), Brief Pain Inventory Short Form (BPI-SF), BPI—Pain, 19-item Functional Assessment of Cancer Therapy—Kidney Symptom Index (FKSI-19), etc. We extracted PROs used, clinical meaningful changes evaluated, and additional statistical methods utilized, as well as results. We aimed to synthesize the available data regarding the impact of these treatments on PWB, functional well-being (FWB), EWB, and SWB, as well as overall life satisfaction of patients receiving these treatments.

Finally, we conducted a separate literature review using PubMed, Embase, and the Cochrane Library to identify studies related to treatment burden, PROs, and QoL in these GU malignancies using search terms including “treatment burden,” “patient-reported outcomes,” “PRO,” “quality of life,” “genitourinary cancers,” “cancer, genitourinary,” and “prostate/bladder/kidney cancer.” We included studies published from 2000 to June 1, 2024, written in English, involving adult human individuals only (age ≥18 yr). Abstracts were screened for relevance, and data regarding treatment burden and impact on QoL were extracted and synthesized.

## 3. Results

### 3.1. Change in life expectancy for patients with prostate, bladder, and kidney cancers since 2000

Despite an increasing incidence of GU malignancies worldwide, cancer-specific mortality rates are declining in many countries (Table 1, example of the USA) [3,13,14]. Supplementary Table 1 presents the summary of changes in OS from the identified phase 2 or 3 trials on prostate, bladder, and kidney cancer.

### 3.2. Bladder cancer

The BA06 30894 trial demonstrated that, in localized muscle-invasive bladder cancer (MIBC), neoadjuvant chemotherapy (NAC) improved OS by a median of 7 mo, with a 16% reduction in the risk of death (Supplementary Table 1) [15]. Simultaneously, new combination therapies have resulted in dramatic changes in life expectancy in patients with advanced urothelial carcinoma (UC), as strikingly demonstrated by the EV-302 trial, which demonstrated that the combination of enfortumab vedotin (EV) and pembrolizumab (P) improved OS by 15.4 mo compared with cisplatin-based chemotherapy in advanced or metastatic UC (HR 0.47; Supplementary Table 1) [16].

### 3.3. Prostate cancer

Since 2000, prostate cancer (PCa) mortality rates have declined in most countries, despite a relatively stable or an increasing incidence rate (Table 1) [17]. This decline may reflect a stage shift in PCa related to increasing screening adoption and risk-adapted treatment, and/or improve multiagent therapy with activity in the castration-sensitive and castration-resistant metastatic spaces [17,18]. Notably, in the metastatic PCa space, the addition of docetaxel increased the median OS by 13.6 mo in the CHAARTED trial [19], while the addition of abiraterone acetate increased the median OS by 16.8 mo in the LATITUDE trial [20]. In patient with metastatic castration-resistant PCa, multiple trials have reported improved median OS of approximately 3–5 mo (see Supplementary Table 1).

### 3.4. Kidney cancer

In metastatic renal cell carcinoma (mRCC), the introduction of tyrosine kinase inhibitors (TKIs) followed by combination therapy with immunotherapy (IO) has resulted in substantive gains in life expectancy and, in some cases, complete response rate, with comparative reductions in toxicity relative to previous cytokine-based treatments (eg, interferon or interleukin-2; Supplementary Table 1). For instance, the median OS for patients treated with sunitinib (a TKI) was 26.4 mo compared with 21.8 mo for those treated with IFN-alpha [21]. More recently, the CheckMate-214 trial reported median OS of 53 mo for the combination of nivolumab and ipilimumab versus 37 mo for sunitinib in first-line mRCC (Supplementary Table 1) [22].

### 3.5. HrQoL—impact of the disease and treatment

#### 3.5.1. Bladder cancer

Bladder cancer (BCa) impacts HrQoL significantly, with reported significant declines in PWB, mental well-being, and SWB relative to controls [23]. For patients who do not receive definitive treatment, BCa inevitably leads to loss of functional and social aspects of QoL [24]. A retrospective study of octogenarians with MIBC treated with radical cystectomy (RC) or transurethral resection of a bladder tumor (TURBT) alone showed that while both groups showed low baseline HrQoL scores, RC patients reported significant improvements in PWB, EWB, and SWB 6 mo after surgery, underscoring the potential benefits of surgery in carefully selected patients [25]. Notably, limited data are available regarding return to QoL and functional independence in this cohort, factors that may be highly relevant to older adults facing the decision regarding RC [26].

The impact of BCa on HrQoL varies across patients, influenced by age and performance status among other risk factors. Catto et al [27] evaluated HrQoL (EQ-5D, EORTC QLQ-C30, BLM30, and NMIBC24) in 1796 patients with BCa, and compared with general population and other pelvic cancer patients. They reported that HrQoL is relatively independent of disease stage, treatment, and multimodal care, but significantly associated with age, comorbidities, sexual function issues, and financial concerns. Younger patients reported more financial difficulties, especially those receiving RC. Generic HrQoL outcomes were similar across disease stages and treatments, but BCa patients experienced worse HrQoL than those with colorectal cancer or PCa and the general population, particularly in mobility and usual activities. In a prospective UK study looking at HrQoL in the 1st year after BCa diagnosis, patients receiving RC (*n* = 51) often reported prolonged declines in mobility, social functioning, and body image compared with those treated with TURBT ± intravesical therapy (*n* = 238), who may see quicker HrQoL recovery to baseline levels [28]. In a 2-yr longitudinal evaluation of PROs following RC, Clements et al [29] reported that by 24 mo, HrQoL returned to or exceeded baseline in all domains other than sexual function, with more body image impact with ileal conduit. Despite these differences, both radical and conservative treatments can result in significant QoL challenges, highlighting the need for individualized treatment planning and comprehensive patient counseling.

There is a lack of reporting measures of HrQoL for most older RCTs investigating the use of NAC prior to RC for localized MIBC. To our knowledge, only JCOG0209 [30] reported a detailed article on the HrQoL assessments in this trial comparing two cycles of methotrexate, vinblastine, Adriamycin, and cisplatin (MVAC) with a control group. They reported that NAC before RC may temporarily worsen PWB and FWB as well as worsen weight loss, diarrhea, appetite, and body appearance initially, but these effects often normalized 1 yr after treatment, suggesting a transient QoL decrement primarily related to treatment phases rather than long-term outcomes (Supplementary Table 2) [30]. Other studies have similarly demonstrated that chemotherapy is associated with transient declines in HrQoL that recover following completion of treatment, including its use in trimodal therapy [31]. Finally, patients receiving gemcitabine and cisplatin perhaps fared better regarding weight, performance status, and fatigue than those receiving MVAC, with a similar survival advantage [32].

Regarding HrQoL during treatment for advanced UC, EV-302 demonstrated transient worsening of global health status and after 3 wk of EV-P, with return to baseline at week 4, which then stabilized through week 26 (−6.3). By comparison, patients treated with cisplatin-based chemotherapy demonstrated deterioration from week 1, with return to baseline by week 17 (range −1.2 to −7.1). Furthermore, patients with moderate to severe pain at baseline treated with EV-P (*n* = 128, 34%) reported a meaningful improvement in pain from weeks 3 through 26 (Supplementary Table 2) [33]. Similarly, patients with advanced UC treated in EV-103 with EV-P or EV monotherapy endorsed preserved or improved QoL, functioning, and/or symptoms (EORTC QLQ-C30), and improvement of pain (BPI-SF) [34]. One critical concern in the literature stems from lacking repeated-measure PRO/HrQoL data, for example, as reported in the CheckMate-901 trial due to low completion rates of PRO questionnaires after 10 wk (Supplementary Table 2) [35]. This highlights the importance of developing protocols prioritizing the completion of HrQoL evaluations and developing parsimonious assessments to limit survey fatigue and reduce the risk of incomplete data collection.

#### 3.5.2. Prostate cancer

PSA screening is associated with improvements in PCa-specific survival [36]. In shared decision-making, the impact of PCa screening and its treatments, such as radical prostatectomy or radiation therapy, on QoL is reviewed alongside discussions potential for PCa progression, risks of cancer-specific mortality, and the QoL implications of advanced PCa. A study by Zheng et al [37] used the Surveillance, Epidemiology, and End Results—Medicare Health Outcomes Survey data linkage to compare HrQoL between PCa survivors aged ≥65 yr (local regionalized, *N* = 19 583; metastatic, *N* = 752) and men without a cancer history (*N* = 782 305). Patients with metastatic PCa reported significantly lower general, physical, and mental health scores than men without a cancer history. By contrast, survivors with nonmetastatic disease reported similar HrQoL scores to those without a cancer history. This work highlights the potential benefits of diagnosing and offering definitive treatment to avoid progression to metastatic disease, as well as the critical need for interventions to support and enhance HrQoL in men with advanced and metastatic PCa.

Multiple trials for advanced PCa have reported improvements in pain (BPI-SF), and either no significant change or improvements in FACT-P scores or subdomains with the investigational treatment compared with the control arm (Supplementary Table 2). Control, delay, or elimination of pain is commonly used as a central PRO in most analyses, in line with the reported most prominent and debilitating symptom associated with castration-resistant PCa [38]. Overall, novel therapeutics seems to be associated with stable QoL or improvement if disease-related toxicity is better controlled (Supplementary Table 2). Some more complex interpretations, however, arise with earlier treatment intensification (eg, in the adjuvant setting). For instance, QoL was evaluated in the EMBARK trial, which compared androgen deprivation therapy (ADT) plus enzalutamide with ADT alone for high-risk biochemical recurrence [39]. Time to first/confirmed clinically meaningful deterioration in QoL favored luteinizing hormone–releasing hormone (LHRH) monotherapy over combination therapy in multiples subdomains (PWB, EWB, FACT-G total, and FACT-P trial outcome indices). Similarly, more subdomains favored LHRH monotherapy over enzalutamide monotherapy (PWB, EWB, PCa pain subscale score, FACT-P trial outcome index, and FACT advance prostate symptoms index). However, this was not significant in the study-defined primary outcome (FACT-P total score and BPI-SF item 3). The conclusion from the authors was that treatment combination, or enzalutamide monotherapy, preserved HrQoL [39].

#### 3.5.3. Kidney cancer

Patients with advanced renal cell carcinoma (RCC) or mRCC report low symptom burden and only slightly lower baseline health status compared with healthy populations [40]. However, psychological distress is reported as an important factor affecting QoL in RCC [41]. In a study from Bergerot et al [42], 151 patients with mRCC (treated with IO, IO-TKI, TKI, or no treatment) reported that worry or fear of disease progression and pain were more frequently attributed to the cancer itself. By comparison, symptoms frequently attributed to treatment included fatigue, lack of energy, diarrhea, appetite, and nausea. Importantly, assessments of patient’s distress related to concerns about their disease recurrence are rarely captured [42].

In KEYNOTE-564, P improved OS compared with placebo for adjuvant therapy following resection of locally advanced non-mRCC [40], a first for adjuvant treatment of RCC. Compared with placebo, P was associated with a higher incidence of any and grade 3/4 serious adverse events (20.7% vs 11.5% and 18.6% vs 1.2%, respectively), with variable duration of adverse events (median 2.9 mo). However, investigators reported no significant differences in HrQoL between arms (Supplementary Table 2) [43]. Treatment intensification with combination therapy for mRCC has demonstrated varied impact on QoL. Overall, IO/TKI combination therapies showed less significant declines in HrQoL than single-agent sunitinib (see Supplementary Table 2 for more detail) [44–47]. However, in a real-world study comparing HrQoL data in 152 patients with mRCC from high-volume medical oncology practices with that in the general American population, respondents had significantly lower HrQoL scores, with no significant difference across treatments [48].

### 3.6. Treatment burden

The Global Burden of Disease (GBD) initiative has quantified the impact of diseases, injuries, and risk factors on populations globally. Using metrics such as disability-adjusted life years (DALYs), which combines years of life lost and years lived with disability, GBD offers a broad overview of disease burden (some results presented in Table 1) [2]. Interestingly, despite the increased ASIRs, both the age-standardized mortality rate and DALYs decreased for PCa and BCa [3]. The GBD provides a valuable wide overview of disease burden, but it may fall short in capturing the complexities and nuances of treatment burden in specific cancers. Indeed, treatment burden is defined as a dynamic process that can change across the course of disease and episodes of care, influenced by numerous factors including age, medical history, and the nature of their treatment [49,50]. It encompasses financial toxicity, time and travel required for care, “workload” requiring resources management, and medication needs [51,52]. Table 2 provides an overview of these burdens and potential mitigation strategies to employ.

Extended life expectancy often leads to increased aging-related multimorbidity. A scoping review synthesizing evidence from eight standardized questionnaires measuring treatment burden in patients with multimorbidity including 24 validation studies and 48 applicative studies demonstrated notable gaps in the data attained on questionnaires, reflecting suboptimal or underinvestigated measurement properties, insufficient development, and unclear rationale for categorizing and interpreting raw scores [53]. Additionally, they reported that many of the tools described were developed and validated in populations with high socioeconomic status, highlighting the need for further research and refinement to effectively measure treatment burden in diverse populations [53]. Unfortunately, the oncology-specific literature often fails to consider management of comorbidities as a source of treatment burden in people with cancer [52], despite a high prevalence of multimorbidity [54,55], and reports that it can increase the complexity of medical management and self-management [56,57].

Few studies have examined how frailty influences oncological treatment burden in older adults. In a cross-sectional survey of 119 patients with non–MIBC, the association between a geriatric assessment–derived Deficit Accumulation Index (DAI) of frailty and patient-reported treatment burden was assessed using the Treatment Burden Questionnaire [58]. The authors reported that increasing DAI was associated with greater patient-reported treatment burden. However, the perceived burden varied among robust and prefrail/frail patients, with prefrail/frail patients reporting more challenges due to self-management tasks and urinary symptoms. By contrast, robust patients tend to be younger and reported more challenges due to cancer treatment side effects, emotional burdens due to cancer worry, and time spent in appointments [58].

## 4. Discussion

### 4.1. Patients are living longer with GU cancers, but are they living well?

In oncology, the imperative to extend survival must be balanced with consideration of treatment burden and HrQoL during treatment and throughout survivorship. While clinical trials typically prioritize survival outcomes, there is a critical need to assess whether the current HrQoL methodology adequately captures meaningful changes in QoL as well as the patient’s lived experience during and after therapy. Routinely employed QoL questionnaires do not universally capture treatment burden and may inadequately characterize the long-term impact of therapy- or cancer-related toxicities. This gap may be particularly important in adjuvant settings or trials where survival gains are more robust, resulting in patients suffering unwanted treatment burden and adverse effects without proportional survival benefits. With gains in life expectancy due to novel treatments, achieving a balance between extending life and preserving quality becomes increasingly crucial. Thus, understanding the short- and long-term tradeoffs of therapy represents the critical knowledge needed to inform shared decision-making conversations around treatment choices.

In this review, we highlight recent advancements in oncological treatments that have revolutionized the management of GU malignancies with substantial gains in OS. However, the impact of therapies on patients and their families is often underestimated with standard clinical reporting, failing to fully capture the complexity of treatment burden. We also recognize the limitations of this narrative review, especially given concerns around the generalizability of available clinical trial results, which may be limited due to strict inclusion criteria as well as implementation gaps [59]. Further limitations include heterogeneity of publications, variability in PROMs, and QoL assessments between trials, which preclude comparisons, lack of systemic review, and formal assessment of the risk of bias or quality assessment. Additionally, given space constraints, we were unable to assess the impact of treatment burden in other GU malignancies, including testicular cancer and penile cancer, for which this topic is also highly salient.

Our understanding of the impact of treatment burden on QoL remains constrained by the quality and relevance of available data in cancer clinical trials. Despite established guidelines such as CONSORT-PRO [30] and SISAPRO-QOL [60,61], we note persistent significant gaps in the reporting, analysis, and interpretation of PROs, especially in trials involving newer therapies. For instance, Safa et al [62] found that less than half of trials leading to FDA approval of IO drugs reported PRO data, with many delays and key deficiencies such as missing hypotheses, inadequate statistical controls, and reliance on descriptive statistics. None of these trials met the SISAPRO-QOL recommendations fully. Furthermore, as treatment options expand, patients often undergo multiple lines of therapy—potentially exposing them to cumulative toxicities from various treatments over time. The implications of these additive toxicities for HrQoL are not well understood. Additionally, the relevance of PROMs is critical; Bergerot et al [42] highlighted that many questions in commonly used instruments (FKSI-19) were not pertinent for patients with mRCC treated with contemporary therapies, indicating a need for refinement and inclusion of the patient perspective in PRO design.

While incorporation of PRO endpoints adds complexity to trial development due to challenges in design, data collection, and handling of missing data [60], doing so is essential to improve the understanding of the real-world impact of cancer treatments on well-being and function, and potentially improve HrQoL [63] and even survival [64]. Recommendations to enhance PROM and QoL assessments include adhering to established guidelines such as SISAQOL/-IMI [60,61] for comprehensive design, analysis, and interpretation of PRO data; SPIRIT-PRO [65,66] for protocol recommendations in clinical trials; or CONSORT-PRO [67] for improving PRO reporting. Examples of key steps include defining PROs clearly as primary or secondary outcomes in the abstract, articulating hypotheses and identifying relevant domains, using validated instruments with detailed methodology and citations, implementing robust statistical plans for handling missing data, and discussing thoroughly the limitations and implications of the results for possible generalizability [67]. Additionally, active endorsement by scientific journals, clinical trial groups, and professional associations, as well as collaboration with advocacy groups is crucial. Improved rigor and transparency in designing, implementing, and reporting of PROs, supported by these stakeholders, are essential to generate high-quality evidence and influence clinical practice guidelines. Finally, developing and validating questionnaire tailored to capture treatment burden, especially in the context of GU malignancies, would be a critical next step.

The current landscape of oncological treatment is characterized by competing trends toward therapy *intensification*, often combining different therapeutics with synergistic mechanisms, as well as moving advanced therapeutics into earlier lines of therapy or in adjuvant setting, and *deintensification*, reflecting the challenges of working to optimize survival while mitigating treatment-related impacts and treatment burden, reducing treatment-related toxicity while maintaining efficacy. Active surveillance protocols for low-risk PCa or bladder-sparing approaches [68,69] endeavor to minimize radical interventions when feasible. Ongoing trials such as A-DREAM (NCT05241860) and EORTC GUCG 2238 (NCT05974774) explore the benefits of dose adjustments and treatment pauses in metastatic PCa to mitigate long-term treatment burdens. Additional upcoming approach of personalized medicine is leveraging genomic and biomarker analyses to tailor therapies precisely to individual patients. Trials such as IMvigor011 (NCT04660344), ALLIANCE A032103, and GUSTO (ISRCTN17378733) will investigate the potential of genomic biomarkers or RNA subtyping to refine patient selection and outcomes to treatment to which they may have increased susceptibility while potentially reducing exposure and toxicities.

Goal-aligned treatment decision-making is a central component of contemporary oncology practice. This approach mandates that we both elicit patient priorities regarding short- and long-term outcomes and collect data regarding patient-centric outcomes such as treatment burden, financial toxicity, as well as detailed data regarding the impact of therapies on HrQoL, to holistically counsel patients regarding the expected outcomes of different therapeutic options. Individuals differentially weigh the factors that influence treatment decision, spanning the likelihood of disease cure, avoidance of toxicity, maintenance of independence, and HrQoL. Addressing individual priorities is essential to foster clinician-patient trust and confidence in treatment outcomes, and to avoid decisional regret. Resources such as decision aids and decision coaches may facilitate priority elicitation and facilitate the shared decision-making process to optimize the likelihood of priority-concordant care [70–72].

## 5. Conclusions

In conclusion, as we achieve advances in extending life expectancy across GU malignancies through innovative oncological treatments, it is imperative to concurrently characterize the implications during and after these treatments on quantity of life and QoL in reproducible and comparable terms, and also to consider how we can work to mitigate treatment burden. Future efforts should focus on refining measurement tools for treatment burden, and ensuring proper incorporation of PROMs in clinical trial design, implementation, and reporting; embracing personalized medicine approaches; and fostering patient-centered decision-making to optimize care outcomes for patients with GU malignancies.

## Supplementary Material

Supplementary Table 1: Summary of change in median overall survival from selected major clinical trial in bladder, kidney and prostate cancer

Supplementary data

Supplementary data to this article can be found online at https://doi.org/10.1016/j.eururo.2024.11.026.

## Figures and Tables

**Table 1 –:** Age-standardized mortality rate per 100 000 in the USA and globally

Cancer	Age-standardized mortality rate per 100 000 males in the USA [13]	Age-standardized mortality rate per 100 000 females in the USA [13]	Age-standardized mortality rate per 100 000 of both sexes globally

Prostate	1990: 18.6	–	2019: 6.32 [3]
	2017: 8.4	–	2022: 7.3 [14]
Kidney	1990: 3.8	1990: 1.7	2019: 2.08 [3]
	2017: 3.0	2017: 1.3	2022: 1.5 [14]
Bladder	1990: 3.8	1990: 1.2	2019: 2.94 [3]
	2017: 3.4	2017: 1.0	2022: 1.8 [14]

**Table 2 –:** Summary of treatment burden domains in oncology

Burden	Consideration and impact	Examples of mitigation strategies

Financial burden	1. Dominant determinant of treatment burden [49] 2. Varies by country, health care system, disease stage, severity, and treatment [49] 3. Increased with minority [51,73] 4. Includes indirect costs such as appointment-related expenses, medical complications, time off work, job loss, or early retirement [51,74,75] 5. Critical linkages between financial toxicity and worse HrQoL [76,77] and social or mental well-being [78,79]	1. De-escalation of visit intensity 2. Grouping visits 3. Developing options for sustained delivery of therapeutics [80] 4. Home-based therapies [80] 5. Utilizing telehealth to save on travel costs [81] 6. Alternative strategies for psychological health delivery (home based, such as expressive writing interventions or online self-management interventions) [41]
Time and travel burden	1. Total time spent traveling to/from medical facilities, waiting for treatments, and duration of treatments [82] 2. Significant impact on QoL, employment, and daily routines [80] 3. Greater burden for patients in rural/remote areas [81] 4. May impact ability to afford healthy nutrition [52]	1. Telehealth [81] 2. Home-based therapies [41,80] 3. Alternative treatment administration (subcutaneous vs intravenous) [83,84] 4. Slow delivery mechanism or longer interval between treatments
“Workload” and resource management	1. Include time and finances 2. Knowledge and skills (communication, ability to find and understand health information, some treatment specific–stoma appliance, etc.) 3. “Energy” to perform daily activity or treatment related	1. As above 2. Importance of social network [52] 3. Delegation of certain tasks [52] 4. Improved translation services and patient education tools
Medication burden	1. Adverse events from antineoplastic medical therapy (diarrhea, fatigue, pain, hot flashes) and impact on daily activities [85] 2. Drug cost may prohibit access or coverage of treatment [86] 3. Current gap in assessing long-term medication-induced treatment burdens such as neuropathy	1. Dose modifications or drug holidays to reduce adverse events [87]

HrQoL = health-related quality of life; QoL = quality of life.

## ADVANCING PRACTICE

### What does this study add?

Future clinical trials of novel therapeutics in genitourinary oncology should prioritize integration of robust patient-reported outcome measures and patient-centered outcomes including treatment burden to support alignment of cancer care with patient priorities, resources, and well-being.

### Clinical Relevance

Quality of life is becoming increasingly important in the management of genitourinary malignancies, particularly as advancements in therapies continue to extend survival. This narrative review highlights the critical gaps in clinical trials on prostate, bladder, and kidney cancer, where measures of patient-centric outcomes, such as treatment burden, remain underreported. Incorporating patient-reported outcomes into clinical trial designs is essential to capture the real-world impact of cancer treatments on physical well-being, daily functioning, and overall quality of life. Although integrating patient-reported outcomes adds complexity to trial design, data collection, and management, it is a necessary step toward aligning therapeutic strategies with individual patient goals and values. By prioritizing comprehensive assessment of treatment burden, clinicians can better support shared decision-making and ensure that the gains in survival are accompanied by meaningful improvements in patients’ lived experiences. This patient-centred approach is vital to advancing holistic cancer care and addressing the evolving needs of patients living with genitourinary malignancies. Associate Editor: Gianluca Giannarini MD.

### Patient Summary

In this article, we reviewed how treatment affects quality of life (QoL) for patients with prostate, bladder, and kidney cancers. We found that, while new therapies improve overall survival, few clinical trials measure the impact of treatment on QoL, and patient-centric outcomes such as treatment burden are captured rarely. Our findings highlight the need for careful measurement of patient-reported outcomes to support shared decision-making around potential tradeoffs and impact of treatments on short- and long-term QoL.
