# Supplementary material for: Increasing Life Expectancy in Patients with Genitourinary Malignancies: Impact of Treatment Burden on Disease Management and Quality of Life
Source: Eur Urol. Author manuscript; Available in PMC 2026 Jul 1. (PMC13097184; doi:10.1016/j.eururo.2024.11.026)
Supplement: Supplementary Table 1: Summary of change in median overall survival from selected major clinical trial in bladder, kidney and prostate cancer [file NIHMS2153976-supplement-Supplementary_Table_1__Summary_of_change_in_median_overall_survival_from_selected_major_clinical_trial_in_bladder__kidney_and_prostate_cancer.docx]

Supplementary Table 1: Summary of change in median overall survival from selected major clinical trial in bladder, kidney and prostate cancer

| **Study** | **Comparison Treatment** | **Investigational Treatment** | **Change in Overall Survival (OS)** |
| --- | --- | --- | --- |
| **Bladder Cancer – Localized Disease** | | | |
| BA06 30894 trial [1] | (no neoadjuvant) | neoadjuvant cisplatin, methotrexate, and vinblastine x 3 | Improved median OS: **+ 7 months** (Median OS: 37 and 44 months)  16% reduction in the risk of death |
| VESPER[2] | Gemcitabine-cisplatin (GC) x 4 | ddMVAC x 6 | 5-year OS 66% (95% CI 60-73) vs 57% (95% CI 50-64), ddMVAC vs GC group, respectively  29% reduction in risk of death (95% CI 0.52-0.97) |
| **Upper Tract Urothelial Carcinoma** | | | |
| POUT trial [3] | Observation | Adjuvant platinum-based chemotherapy | Improved OS: The 5-year OS 66% vs 57% (platinum chemotherapy vs observation, respectively), with univariable HR, 0.68 (95% CI, 0.46 to 1.00, P = .0049).  Restricted mean survival time difference **11 months** (78 and 67 months, 95% CI, 1 to 21, P = .036) |
| **Advanced or metastatic bladder cancer** | | | |
| von der Maase et al (RCT chemotherapy) [4] | GC | MVAC | Median OS similar in both arms (14 vs 15.2 months for GC vs MVAC, respectively, HR: 1.09; 95% CI, 0.88 to 1.34; P = .66) |
| EV-302 trial [5] | Cisplatin-based | EV-P | Improved median OS: **+ 15.4 months.** (31.5 months vs 16.1 months with EV-P vs chemotherapy, respectively)  53% reduction in risk of death |
| CheckMate 901 [6] | Cisplatin-based | GC + Nivolumab | Improved median OS: **+ 2.8 months** (21.7 months [95% CI, 18.6-26.4) vs 18.9 months [95% CI, 14.7-22.4] for GC + Nivolumab vs Cisplatin-based chemotherapy, respectively) |
| **Localized Kidney – Adjuvant Treatment** | | | |
| KEYNOTE-564  [7] | Placebo | Adjuvant Pembrolizumab | Improved OS: 4-year OS 91.2% vs 86% (Pembrolizumab vs placebo, respectively)  38% reduction in risk of death (95% CI, 0.44 to 0.87; P=0.005). |
| **Advanced or Metastatic RCC** | | | |
| Motzer et al., 2009[8] | IFN-alpha | Sunitinib | Improved median OS: **+ 4.6 months. (**26.4 vs 21.8 months for sunitinib vs IFN-alpha, respectively) |
| CLEAR trial[9] | Sunitinib | Lenvatinib plus pembrolizumab | Improved OS: Median 53.7 months (95% CI, 48.7 to not estimable [NE]) with lenvatinib plus pembrolizumab versus 54.3 months (95% CI, 40.9 to NE) with sunitinib; respectively  21% reduction in risk of death (HR 0.79 (95% CI, 0.63 to 0.99; nominal P value = .0424)) |
| Keynote-426 [10] | Sunitinib | Pembrolizumab plus axitinib | Improved median OS: **+ 6 months (**46 vs 40 months for Pembrolizumab + axitinib vs sunitinib, respectively).  27% reduction in risk of death (HR 0.73, 95% CI, 0.60–0.88) |
| CheckMate 9ER [11] | Sunitinib | Nivolumab plus cabozantinib | Improved median OS: **+ 14** months. (49.5 vs 35.5 months for Nivolumab + Cabozantinib vs sunitinib, respectively). HR 0.60, 95% CI (0.44-0.82) |
| CheckMate214 [12] | Sunitinib | Nivolumab plus ipilimumab | Improved median OS: **+ 16 months**. (53 [46-65] vs 37 months [32-44] for Nivolumab + Ipilimumab vs sunitinib, respectively)  28% reduction in risk of death (HR 0.72, 95% CI 0.62-0.83) |
| **Prostate – non-Metastatic Castration Sensitive Prostate Cancer** | | | |
| EMBARK [13] | ADT alone (leuprolide) | Enzalutamide + ADT | Interim reported 5-year OS 92.2% (95% CI, 88.7 to 94.7) vs 87.2% (83.0 to 90.4) for Enzalutamide + Leuprolide vs Leuprolide monotherapy, respectively  41% reduction in risk of death 0.59 (95% CI, 0.38 to 0.91; p=0.02 [interim efficacy boundary, P≤0.0001]). |
| **Metastatic, Castration Sensitive Prostate Cancer** | | | |
| CHAARTED [14] | ADT alone | Docetaxel + ADT | Improved median OS for high-volume disease: **+ 13.6 months** (51.2 vs 34.4 months for Docetaxel + ADT versus ADT monotherapy, respectively)  37% reduction in risk of death (95% CI, 0.50 to 0.79; p < .001) |
| Latitude [15] | ADT + placebo + prednisone | ADT+ abiraterone acetate + prednisone | Improved median OS: **+ 16.8 months** (53.3 [95% CI 48.2–not reached]) vs 36.5 months [33.5–40.0] for Abiraterone and prednisone + ADT vs ADT, respectively)  34% reduction in risk of death (95% CI 0.56–0.78; p<0.0001). |
| Arasens [16] | ADT + docetaxel + placebo | ADT + docetaxel + Darolutamide | 4-year OS 62.7% (95% CI, 58.7 to 66.7) vs 50.4% (95% CI, 46.3 to 54.6) for Darolutamide + Docetaxel + ADT vs placebo + Docetaxel + ADT, respectively  32.5% reduction in risk of death (HR 0.68; 95% CI: 0.57 to 0.80; p<0.001) |
| **Metastatic, Castration Resistant Prostate Cancer** | | | |
| TAX 327 [17] | Mitoxantrone | Docetaxel | Improved median OS: **+ 2.4 months** (18.9 vs 16.5 months for Docetaxel vs Mitoxantrone, respectively)  24% reduction in risk of death (95% CI, 0.62 to 0.94; P=0.009). |
| COU-AA-301 [18] | Prednisone + placebo | Abiraterone acetate + prednisone | Improved median OS: **+ 4.6 months** (15.8 [95% CI 14.8-17.0] vs 11.2 months [10.4-13.1] for Abiraterone acetate + prednisone versus Placebo + prednisone, respectively)  26% reduction in risk of death (95% CI 0.64-0.86; p<0.0001) |
| Affirm[19] | ADT + placebo | ADT + enzalutamide | Improved median OS: **+ 4.8 months** (8.4 (95% CI 17.3 – not yet reached) vs 13.6 months (95% CI, 11.3 to 15.8) for Enzalutamide vs placebo, respectively  37% reduction in risk of death (95% CI, 0.53-0.75; P<0.001) |
| PROfound [20, 21] | ARPI switch + ADT | Olaparib + ADT | Improved median OS: + 5.7 months for BRCA alteration treated with Olaparib. All cohort; median OS **+ 4.8 months**. |
| PROpel [22] | Abiraterone + pred+ADT + placebo | Olaparib + Abiraterone + pred+ADT | Improved median OS for ITT population: **+ 7.4 months** (42.1 vs. 34.7 months for Olaparib + Abiraterone and prednisone + ADT vs Placebo + Abiraterone and prednisone + ADT, respectively)  18% reduction in risk of death (95% CI 0.67-1.00; p=0.054 |

ADT: androgen deprivation therapy; ARPI: androgen receptor pathway inhibitor; CI: confidence interval; ddMVAC: Dose-Dense Methotrexate, Vinblastine, Doxorubicin, and Cisplatin; EV-P: enfortumab-vedotin combined with pembrolizumab; GC: Gemcitabine and Cisplatin; HR: Hazard Ratio; IFN: interferon; ITT: intention to treat; MVAC : Methotrexate, Vinblastine, Doxorubicin, and Cisplatin; OS: overall survival.

Supplementary Table 2: Summary of quality of life and patient reported outcome from selected major clinical trial in bladder, kidney and prostate cancer

| **Study** | **Comparison Treatment** | **Investigational Treatment** | **QOL and PRO use, and threshold** | **Summary of QoL and PRO** |
| --- | --- | --- | --- | --- |
| **Bladder Cancer – Localized Disease** | | | | |
| JCOG0209 [23] | No neoadjuvant | MVAC x 2 | FACT-Bl. Used interpretation manual: threshold 2-3 for subscale, and 5-7 for FACT-G total). | Lower (worst) scores at the time-point post-NAC for PWB, FWB, FACT-G total, weight loss, diarrhea, appetite, body appearance, embarrassment by ostomy appliance and total FACT-Bl. However, there was no difference in scores for these domains between the two arms after radical cystectomy and 1 year after registration, except for except for embarrassment by ostomy appliance. |
| VESPER[2] | GC x 4 | ddMVAC x 6 |  | No PRO reported |
| BC2001 [24] | Bladder radiation | Trimodal therapy (chemo-radiation of the bladder) | FACT-Bl. Individual comparison (comparison of the percentage of participants experiencing a minimal clinically significant change from baseline) and between group comparison. Minimal clinically significant change: defined as a 3-point change in bladder cancer subscale, 5-point in TOI or 7-point in total score. | Patients had an initial reported detriment to their QoL (after end of treatment) (bladder cancer subscale -5.06 [99% CI: -6.12 to -4.00, p< 0.001]; overall FACT-Bl Total score -8.22 [-10.76 to -5.68, p< 0.01]), but returned to baseline within six months of treatment with trimodal therapy. The addition of concomitant chemotherapy or neoadjuvant chemotherapy did not significantly impact HrQoL. |
| **Upper Tract Urothelial Carcinoma** | | | | |
| POUT trial [3] | Observation | Adjuvant platinum-based | Adverse event and EORTC-QLQ-C30. Legacy threshold of >10 point for clinically significant change. | CTCAE grade ≥3 rates between 6 and 24 months were similar in both groups (40 of 240, 16.7%).  No important differences in QoL (EORTC-QLQ-C30) were observed between group at 12 and 24 months. Number of enrol participant for QoL analysis was low (72 and 83 participants). |
| **Advanced or Metastatic Bladder Cancer** | | | | |
| EV-302 trial [25]. | Cisplatin-based | EV-P | Pain assesses via BPI-SF item 3. EORTC-QLQ-C30 with meaningful difference: A change of 5 - 10 points is considered a small change. A change of 10 - 20 points is considered a moderate change (from NCT04223856 and abstract only). | Patients with moderate to severe pain at baseline who were treated with EV-P (n=128, 34%) had a meaningful (>2 points) improvement from baseline in BPI worst pain from week 3 through 26. EORTC QLQ-C30 Global Health Status/Quality of Life [GHS/QoL], EV+P demonstrated a transient worsening at week 3 (-6.3) that returned to baseline from weeks 4 through 26, while patients treated with cisplatin-based chemotherapy demonstrated deterioration from week 1 through week 17 (range -1.2 to -7.1) when scores returned to baseline |
| CheckMate 901 [6] | Cisplatin-based | GC with Nivolumab | EORTC QLQ-C30 significant difference define by change of > 10 points between groups. | Low completion rate of forms past 10 weeks. EORTC QLQ-C30 stable and no significant difference. |
| **Localized Kidney - Adjuvant** | | | | |
| KEYNOTE-564  [26] | Placebo | Pembrolizumab | LSM change from baseline to week 52 in symptom scores as assessed by the EORTC QLQ-C30 GHS/QoL and physical functioning scale scores. Clinically meaningful change threshold was considered a ≥ 10-point increase or decrease, and for subscale.  FKSI-DRS was use with a clinically meaningful change threshold defined as a 2- to 3-point change from baseline. Exploratory end points included mean change from baseline in EORTC QLQ-C30 functioning and symptom scales and the EQ VAS (clinically meaningful change threshold defined as a ≥ 7-point change from baseline). | A trend to inferior EORTC QLQ-C30 GHS/QoL but not statistically significant difference in LSM scores for pembrolizumab vs placebo were observed at week 52 for EORTC QLQ-C30 GHS/QoL (–2.5; 95% CI –5.2 to 0.1), EORTC QLQ-C30 physical functioning (–0.87; 95% CI –2.7 to 1.0), and FKSI-DRS (–0.7; 95% CI –1.2 to –0.1). Most PRO scores remained stable or improved for the EORTC QLQ-C30 GHS/QoL (pembrolizumab, 54.3%; placebo, 67.5%), EORTC QLQ-C30 physical functioning (pembrolizumab, 64.7%; placebo, 68.8%), and FKSI-DRS (pembrolizumab, 58.2%; placebo, 66.3%).  Pembrolizumab was associated with a higher incidence of serious adverse events of any cause than placebo (20.7% vs. 11.5%), as well as with a higher incidence of adverse events of any grade (79.1% vs. 53.0%) or of grade 3 or 4 (18.6% vs. 1.2%) |
| **Advanced or Metastatic RCC** | | | | |
| TKI era: [27, 28] | IFN-alpha | Sunitinib | FACT-G and FKSI questionnaires, at randomization, day 1 and 28 of each cycle and at end of treatment.  In Castellano et al.[28], repeated measures mixed-effects models (MEMs) were used to assess the between-treatment differences for all the PRO end points. Estimation of variance by restricted/residual maximum likelihood. LSM for the first 9 cycles for each PRO measure.  Minimal important difference (MID): FKSI: 3-5 points, FKSI-DRS: 2-3 points, FACT-G: N/A, EQ-5D: not applicable. | FKSI-DRS and FKSI scores exhibited statistically significant patterns favoring the sunitinib group over the IFN-a group. Using MEM of FKSI-DRS 9 item, compared with the IFN-a group, patients on sunitinib demonstrated significantly milder symptoms (higher LSM scores) of bone pain, fatigue, and fevers. Compared with the MID, the treatment differences were considered clinically meaningful after cycle 2 day 1 for the FKSI-DRS and FKSI.  Based on the mean treatment difference for the FACT-G scores, patients in the sunitinib group experienced statistically better cancer-specific HrQoL compared with patients in the IFN-a group at each time point. (Also significant if use MID of 5 points after first cycle.)  Based on the LSM of EQ-5D index, patients in the sunitinib group reported better general health status than patients in the IFN-a group. The pattern of EQ-5D utility and EQ-VAS LSM predicted scores was very different, with a faster increasing utility in the INF group. This lack of consistency between EQ scores advises to interpret results with care. |
| CLEAR [29] | Sunitinib | Lenvatinib plus pembrolizumab, or lenvatinib plus everolimus | FKSI-DRS, EORTC QLQ-C30, EQ-5D-3L. at baseline and on day 1 of each subsequent 21-day cycle. Differences in LSM between each lenvatinib treatment group (lenvatinib plus pembrolizumab and lenvatinib plus everolimus) and sunitinib. MID: decrease of 3 or more points for the FKSI-DRS, a decrease of 10 or more points for the EORTC QLQ-C30, a decrease of 0.08 or more points for the EQ-5D-3L index; and a decrease of 7 or more points for the EQ-5D-3L VAS. Death = deterioration. Time to deterioration and time to definitive deterioration (no recovery). | Lenvatinib + pembrolizumab had similar or favourable scores compared with patients given sunitinib. Analyses of time to first deterioration showed a similar outcome between the lenvatinib plus pembrolizumab and sunitinib treatment groups across most instruments; Shorter (worst) median time to first deterioration (9.14 weeks compared to 12.14 weeks for FKSI-DRS) but not significant (HR 1.13 [95% CI 0.94–1.35], log-rank p=0.20).  12 weeks (7.29–15.14) vs 9.14 weeks (6.29–12.14; 0.88 [0.74–1.05], log-rank p=0.17) for EORTC QLQ-C30 GHS/QOL.  LSM change at mean follow-up (46 weeks, cycle 15) from baseline in the lenvatinib plus pembrolizumab group compared with the sunitinib group was –1.75 (SE 0.59) vs –2.19 (0.66) for FKSI-DRS, –5.93 (0.86) vs –6.73 (0.94) for EORTC QLQ-C30 GHS/QOL, and –4·96 (0.85) vs –6.64 (0.94) for the EQ-5D visual analogue scale. |
| Keynote-426 [30] | Sunitinib | Pembrolizumab plus axitinib | EORTC QLQ-C30, FKSI-DRS, EQ-5D-3L.  MID: FKSI-DRS 3-point change from baseline, EORTC QLQ-C30 $\geq$10-point change from baseline  EQ-5D-3L VAS $\geq$7-point change from baseline. PROs were assessed on day 1 of each cycle in the pembrolizumab + axitinib arm (=after treatment), whereas PROs were assessed on days 1 and 29 of each cycle for up to four cycles, and then on day 1 of each subsequent cycle (following the 2-week off treatment period) in the sunitinib arm. Changes from baseline in LSM scores and compared between treatment groups using a constrained longitudinal data analysis (cLDA) model (Liang and Zeger). Missing data: multiple imputation under the missing at random assumption. Time to confirmed deterioration: time from baseline to first deterioration (meeting or exceeding the MID), with confirmation at the next visit. | The primary analysis time point for PRO end points was reached at week 30, which was the last time point when completion and compliance rates were approximately 60% and 80%.  Better or not different overall improvement rates from baseline between pembrolizumab + axitinib and sunitinib were observed for the FKSI-DRS (–0.79% improvement vs sunitinib; 95% confidence interval [CI] –7.2 to 5.6), QLQ-C30 (7.5% improvement vs sunitinib; 95% CI 1.0–14), and EQ-5D VAS (9.9% improvement vs sunitinib; 95% CI 3.2–17).  No difference in time to confirmed deterioration, except FKSI-DRS favoring sunitinib.  By cLDA analysis, LSM changes from baseline to 150 weeks were not different between group for FKSI-DRS and the EQ-5DVAS, although the LSM was higher with pembrolizumab + axitinib than with sunitinib for the QLQ-C30GHS/QoL  Time of assessment in sunitinib (after 2 weeks off treatment) might have help patient reporting better QoL compared to reporting when on treatment. |
| CheckMate 9ER [31] | Sunitinib | Nivolumab plus cabozantinib | FKSI-19, EQ-5D-3L. PRO data were collected from baseline and at each planned visit: more frequently collected in nivolumab + cabozantinib arm (every 2 weeks) compared with those receiving sunitinib monotherapy (every 6 weeks). MID as previously established: FKSI: 5, EQ-5D-3L: 7 with sensitivity assessment using different threshold using a pattern-mixture model. Longitudinal change from baseline was evaluated via mixed-model repeated measures (MMRM) analysis. Death or progression were not considered deterioration events. | Completion rate > 75% up to week 103 in combination, and >75% up to week 79 for sunitinib.  Initial decline in most score in both groups. In MMRM analysis, nivolumab-cabozantinib were stable (FKSI-19 and EQ-5D-3L) or improved (FKSI-19 DRS-v1 and EQ-5D-3L VAS) over time. In contrast, sunitinib decline in all PROs. However, none of the change exceeded the define MID, except deterioration of EQ-5D-3L in the sunitinib after week 55 on multiples time-point.  TTFD/TTCD favored nivolumab + cabozantinib vs sunitinib. Compared with sunitinib, nivolumab + cabozantinib significantly delayed time to deterioration. |
| CheckMate 214 [32] | Sunitinib | Nivolumab plus ipilimumab | FKSI-19, FACT-G, EQ-5D-3L. All PRO instruments were administered on day 1 of week 1 and day 1 of week 4 of the first two cycles; day 1 of week 1 and day 1 of week 5 of the next two cycles; day 1 of week 1 of the subsequent cycles; and at the first two follow-up visits.  Main analysis threshold: FKSI-19: 3, FACT-G: 7, ED-5D-3L VAS: 7 (UK utility index: 0.08).  Mean score and MMRM analysis for longitudinal assessment. | At week 25, there was approx. 50% completion rate in the study population. However, the proportion of participants who completed the assessments (among those expected to complete them) was more than 80% for all PRO instruments at most visit.  Almost all PRO scores after baseline were higher in participants in the nivolumab plus ipilimumab group than in those in the sunitinib group, with some exception staring after 103 weeks. Based on MMRM model data, mean change in FKSI-19 score, FACT-G score, and EQ-5D-3L utility index score was higher with nivolumab plus ipilimumab than with sunitinib at most timepoints assessed, but not for EQ-5D-3L VAS. LSM change in score for all instruments was improved in the nivolumab plus ipilimumab group as early as week 10 and was maintained until week 103.  Mean change FKSI-19 total score being 4.00 (95% CI 1.91 to 6.09) for nivolumab plus ipilimumab vs -3.14 (-6.03 to -0.25) for sunitinib (p<0.0001).  FACT-G total score being 4.77 (1.73 to 7.82) for nivolumab plus ipilimumab vs -4.32 (-8.54 to -0.11) for sunitinib (p=0.0005). |
| **Prostate – non-Metastatic Castration Sensitive Prostate Cancer** | | | | |
| EMBARK [33] | ADT alone + placebo | Enzalutamide + ADT (blinded) or  Enzalutamide monotherapy (open arm) | TTFD/TTCD using FACT-P, EORTC QLQ-PR25 and EQ-5D-5L. Measure at baseline and every 12 weeks until metastasis or death. | Overall, many subdomains of FACT-P favored ADT + placebo compared to enzalutamide monotherapy (PWB, EWB, prostate cancer pain subscale score, FACT-P TOI, FACT advance prostate symptoms index) or ADT alone vs combination therapy (PWB, EMB, FACT-G total and FACT-P TOI), and others were not significant. EORTC QLQ-PR25 subdomain meant criteria for deterioration over time except bowel symptoms/function for all treatment group, but was not a significant difference. Pain, as measure by item 3 of BPI-SF, and FACT-P total score, did not significantly differ between group (primary outcomes: no difference with treatment arm).  Sexual activity appears to be better preserved with enzalutamide monotherapy than with placebo + ADT. |
| **Metastatic, Castration Sensitive Prostate Cancer** | | | | |
| CHAARTED [34] | ADT alone | Docetaxel + ADT | FACT-P; MID of 6–10 points, range 0–156, higher scores indicate better QOL.  FACIT-F; MID of 3 points, range 0–160, higher scores reflect less fatigue  BPI; MID change of 2 points, range 0–10, higher scores indicating worse pain. Measure at baseline and 3, 6, 9, and 12 months, with completion rate of FACT-P surveys of 90%, 86%, 83%, 78%, and 77% respectively. | Between 2 group, there were a decline in FACT-P at 3 months and not significant at 12 months with docetaxel compared to ADT alone. More fatigue at 3 months, but not significant at 12 months  When comparing QoL with OS,  baseline higher QoL by FACT-P (n=705) was associated with better OS in univariate regression (HR 0.70 [0.55,0.90], p = 0.005) (not significant on multivariate analysis). On multivariate analysis, patients with poorest baseline QoL who received ADT + docetaxel had a trend toward improved survival compared with to ADT alone (45.2 vs 34.4 months, HR 0.75 [0.53, 1.05], p = 0.09). |
| LATITUDE [35] | ADT + placebo + prednisone | Abiraterone acetate + prednisone + ADT | BPI-SF, BFI, FACT-P, EQ-5D-5L  Baseline and before any other visit procedure on day 1 of cycles 1–3, monthly during cycles 4–13, and then every 2 months until the end of treatment. Only EQ-5D-5L after treatment discontinuation (up to 12 months)  Outcome: time to average pain progression (MID on BPI-SF items 3–6), Time to worst pain intensity progression (MID from item 3 of BPO-SF, at two consecutive evaluations 4 or more weeks apart).  MMRM.  MID: BPI-SF worst pain: change of $\geq$30% from baseline. BPI-SF pain interference with daily living: change of $\geq5$0% from baseline SD. FACT-P: $\Delta$10-point. FACT-G: $\Delta$9-point, subscale: $\Delta$3-point. BPI worst pain: 2-point. | Compliance was 90% or higher for all PRO measurement tools. With abiraterone acetate, improvement in symptoms (pain, fatigue) and in QoL and longer time to deterioration in functional status (pain (HR 0.63 [95% CI 0.52–0.77]; p<0.0001) and fatigue (HR 0.65 [95% CI 0.53–0.81], p=0.0001)).  Prolonging time to QOL deterioration.  Multiples outcome were not reached at time of publication.  Changes from baseline in worst fatigue intensity and fatigue interference mean scores, (repeated-measures mixed-effect model), were improved with ADT plus abiraterone compared with placebo group as early as cycle 5 and maintained through cycle 33 except at two timepoint (cycle 27 for worst fatigue intensity and cycles 19 and 27 for fatigue interference). |
| ARASENS [16, 36] | ADT + docetaxel + placebo | ADT + docetaxel + darolutamide | Did not publish results from PRO using validated questionnaire other than abstract format and NCCN-FACT-FPSI-17 questionnaire. Only AE, time to AE, time to worsening of disease-related physical symptoms (NCCN-FACT-FPSI-17) | The time to pain progression was significantly longer in the darolutamide group (hazard ratio, 0.79; 95% CI, 0.66 to 0.95; P=0.01). Comparable time to worsening disease-related physical symptoms in both arms. |
| **Metastatic, Castration Resistant Prostate cancer** | | | | |
| TAX 327 [17] | Mitoxantrone | Docetaxel (Every 3 weeks or weekly) | FACT-P and Present Pain Intensity scale from the McGill–Melzack questionnaire. Baseline, every 3 weeks during therapy, and every month after the completion of therapy. QoL response define as +16-point improvement in their FACT-P score, as compared with baseline, on two measurements obtained at least three weeks apart. | N=815 (on 1006 total).  Percentage patient with improvement in QoL similar in the 2 docetaxel regimen group (22% every 3 weeks and 23% for weekly docetaxel) and this was higher than mitoxantrone group (13%) (P=0.009 and P=0.005, respectively). Greatest change was in prostate-specific concerns subscale. |
| COU-AA-301 [37] | ADT + prednisone | ADT + abiraterone acetate + prednisone | FACT-P.  Baseline and on day 1 of cycles 1, 4, 7, 10 and every six cycles thereafter until the end of study treatment.  MID: FACT-P total: $\Delta$10-point.  FACT-G scale and TOI: $\Delta$9-point. Other subscale: $\Delta$3-point  Improvement: Only patients who had impaired HrQoL were considered for the improvement analyses.  Deterioration: patient starting with baseline at least 1 MID above the lower limit.  No imputation of missing assessments was conducted.  Longitudinal: Mean FACT-P total and sub-scale scores were compared between the treatment groups using two models: MMRM models and joint mixed-effects and log time-to-dropout model. | Compliance with questionnaires: 91% at cycle 28 (excluding death)  Significant improvements in the  FACT-P total score was observed in 48% of patients receiving abiraterone vs 32% of patients receiving prednisone (p < 0.0001).  Better FACT-P outcomes with abiraterone, except with SWB which was not significant.  Shorter median time to improvement in PWB and TOI with abiraterone and l onger median time to deterioration.  Mixed-effects model showed that the change in FACT-P total score from baseline was consistently better with abiraterone than prednisone. Again, only SWB not significant.  Joint mixed-effects and log time-to-dropout models suggested that the FACT-P total score profile of abiraterone was superior to that of prednisone (p<0.001) (*higher score = better outcome). All subscale significant except SWB |
| AFFIRM [38] | ADT + placebo | ADT + enzalutamide | FACT-P scores at baseline and at weeks 13, 17, 21, and 25, and every 12 weeks thereafter while under treatment.  No single or certain MID for all applications; range of important differences provided: FACT-P total score, 6–10 points; FACT-P-G total score, 5–7 points; all FACT-P subscales, 2–3 points; TOI, 5–9 points; FAPSI, 2–3 points; and pain-related score, 1–2 points.  Longitudinal: MMRM, missing data assumed at random.  Missing data also assess by pattern mixture model with placebo-based pattern imputation. | N= 938 / 1199 completed baseline + at least 1 other time.  After week 25, FACT-P total score decreased by 1.52 points with enzalutamide compared with 13.73 points with placebo (P < 0.001).  Changes in FACT-P total score and all subscale scores from baseline were significantly smaller (indicating less HrQoL deterioration) with enzalutamide than placebo.  Compared with the pre-established MIDs, the changes observed for all scores in the enzalutamide group were small or negligible, while the changes observed in placebo group were considered clinically meaningful for all scores by week 13 or 17, except for the SWB and EWB domains (= worst QoL).  pattern mixture model and MMRM results similar. |
| PROfound[39] | ARPI switch + ADT | Olaparib + ADT | BPI-SF: 7 days before randomisation and then every 4 weeks thereafter for up to 24 weeks after progression or discontinuation  FACT-P and G: baseline and then every 8 weeks until 24 weeks after progression or discontinuation.  TTPP: time to an increase of ≥2 points in BPI-SF item 3  MID FACT: change of score of 6 or more (FACT-P total score), 5 or more TOI, 3 or more (FAPSI-6, prostate cancer subscale), or 2 or more points (PWB and FWB wellbeing).  MMRM used | Compliance: 90 and 93% (placebo)  Median TTPP significantly longer with olaparib: not reach vs 9.9 months (HR favoring olaparib: 0.44, 95% CI = 0.22–0.91, P = .019)  Differences between the two groups for time to deterioration in FACT-P total score and subscale scores for TOI, FWB, PWB, prostate cancer subscale, and FAPSI-6 were not statistically significant.  LSM change from baseline in BPI-SF pain interference score was significantly lower in the olaparib group than in the control.  % of patient with clinically meaningful improvement in FACT-P total score during treatment was higher for the olaparib group (OR 8.32; 95% CI 1.64–151.84; p_nominal_=0.0065). |
| PROpel [40, 41] | Abiraterone acetate + prednisone + ADT + placebo | Abiraterone acetate + prednisone + ADT + Olaparib | TTPP BPI-SF, FACT-P, EQ-5D-5L.  Baseline and weeks 4, 8, and 12; then every 12 weeks until discontinuation. If discontinuation (for reasons other than disease progression) assessed 30 days after discontinuation, then every 12 weeks until progression.  MID BPI-SF: $\geq$2 points. FACT: $\geq$6 points for FACT-P Total Score; $\geq$ 5 points for TOI; $\geq$3 points for FAPSI-8, FAPSI-6, and PCS; and $\geq$2 points for FWB and PWB. | No difference in % of patient who had not experience pain progression. No meaningful difference in TTPP.  Least-squares mean changes from baseline between arms in BPI-SF pain severity (−0.06; 95% CI −0.23–0.12), pain interference (difference, −0.12; 95% CI −0.31–0.06) worst pain score (difference, −0.12; 95% CI −0.35–0.11) and FACT-P total score (difference, −0.54; 95% CI –3.00–1.92) suggest no clinically meaningful difference in HrQoL between groups. |

ADT: androgen deprivation therapy; AE: adverse event; ARPI: androgen receptor pathway inhibitor; BPI-SF: Brief Pain Inventory–Short Form; CI: confidence interval; CTCAE: common terminology criteria for adverse events; ddMVAC: Dose-Dense Methotrexate, Vinblastine, Doxorubicin, and Cisplatin; EORTC-QLQ-C30: European organisation for research and treatment of cancer - quality of life questionnaire core 30; EORTC-QLQ-PR25: European organisation for research and treatment of cancer - quality of life questionnaire prostate cancer 25; EQ-5D-3L: EuroQol-5D 3-level; EQ-5D-5L: EuroQol-5D-5 level, EQ-VAS: EuroQol visual assessment scale; EMB: emotional wellbeing; EV-P: enfortumab-vedotin combined with pembrolizumab; FACT: Functional Assessment of Cancer Therapy; FACT-Bl: FACT-bladder; FACT-G: FACT-General; FACT-P: FACT-Prostate; FAPSI: FACT Advanced Prostate Symptom Index; FKSI: FACT –Kidney Symptom Index; FKSI-DRS: FACT –Kidney Symptom Index - Disease Related Symptoms; FWB: functional wellbeing; GC: Gemcitabine and Cisplatin; GHS/QoL: Global Health Status/Quality of Life, HR: Hazard Ratio; HrQoL: health-related quality of life; IFN: interferon; LSM: least-square mean; MEMs: measures mixed-effects models; MID: Minimal important difference; MCSCI: McCusker Subjective Cognitive Impairment Inventory; MMRM: mixed-model repeated measures; MVAC: Methotrexate, Vinblastine, Doxorubicin, and Cisplatin; NAC: neoadjuvant chemotherapy; NCCN-FACT-FPSI-17: National Comprehensive Cancer Network/Functional Assessment of Cancer Therapy Prostate Cancer Symptom Index - 17 Item; OS: overall survival; PRO: patient reported-outcome; PWB: physical wellbeing; QoL: quality of life; SWB: social wellbeing; TOI: Trial Outcome Index; TTCD: time to confirmed clinically meaningful deterioration; TTFD: time to first clinical meaningful deterioration; TTP: time to pain progression

**References:**

[1] Griffiths G, Hall R, Sylvester R, Raghavan D, Parmar MK. International phase III trial assessing neoadjuvant cisplatin, methotrexate, and vinblastine chemotherapy for muscle-invasive bladder cancer: long-term results of the BA06 30894 trial. J Clin Oncol. 2011;29:2171-7.

[2] Pfister C, Gravis G, Flechon A, Chevreau C, Mahammedi H, Laguerre B, et al. Perioperative dose-dense methotrexate, vinblastine, doxorubicin, and cisplatin in muscle-invasive bladder cancer (VESPER): survival endpoints at 5 years in an open-label, randomised, phase 3 study. Lancet Oncol. 2024;25:255-64.

[3] Birtle AJ, Jones R, Chester J, Lewis R, Biscombe K, Johnson M, et al. Improved Disease-Free Survival With Adjuvant Chemotherapy After Nephroureterectomy for Upper Tract Urothelial Cancer: Final Results of the POUT Trial. Journal of Clinical Oncology. 2024;42:1466-71.

[4] Von Der Maase H, Sengelov L, Roberts JT, Ricci S, Dogliotti L, Oliver T, et al. Long-term survival results of a randomized trial comparing gemcitabine plus cisplatin, with methotrexate, vinblastine, doxorubicin, plus cisplatin in patients with bladder cancer. Journal of Clinical Oncology. 2005;23:4602-8.

[5] Powles T, Valderrama Begoña P, Gupta S, Bedke J, Kikuchi E, Hoffman-Censits J, et al. Enfortumab Vedotin and Pembrolizumab in Untreated Advanced Urothelial Cancer. New England Journal of Medicine. 2024;390:875-88.

[6] Heijden MSvd, Sonpavde G, Powles T, Necchi A, Burotto M, Schenker M, et al. Nivolumab plus Gemcitabine–Cisplatin in Advanced Urothelial Carcinoma. New England Journal of Medicine. 2023;389:1778-89.

[7] Choueiri TK, Tomczak P, Park SH, Venugopal B, Ferguson T, Symeonides SN, et al. Overall Survival with Adjuvant Pembrolizumab in Renal-Cell Carcinoma. New England Journal of Medicine. 2024;390:1359-71.

[8] Motzer RJ, Hutson TE, Tomczak P, Michaelson MD, Bukowski RM, Oudard S, et al. Overall survival and updated results for sunitinib compared with interferon alfa in patients with metastatic renal cell carcinoma. J Clin Oncol. 2009;27:3584-90.

[9] Motzer RJ, Porta C, Eto M, Powles T, Grünwald V, Hutson TE, et al. Lenvatinib Plus Pembrolizumab Versus Sunitinib in First-Line Treatment of Advanced Renal Cell Carcinoma: Final Prespecified Overall Survival Analysis of CLEAR, a Phase III Study. Journal of Clinical Oncology. 2024;42:1222-8.

[10] Plimack ER, Powles T, Stus V, Gafanov R, Nosov D, Waddell T, et al. Pembrolizumab Plus Axitinib Versus Sunitinib as First-line Treatment of Advanced Renal Cell Carcinoma: 43-month Follow-up of the Phase 3 KEYNOTE-426 Study. European Urology. 2023;84:449-54.

[11] Choueiri TK, Powles T, Burotto M, Escudier B, Bourlon MT, Zurawski B, et al. Nivolumab plus Cabozantinib versus Sunitinib for Advanced Renal-Cell Carcinoma. N Engl J Med. 2021;384:829-41.

[12] Tannir NM, Escudier B, McDermott DF, Burotto M, Choueiri TK, Hammers HJ, et al. Nivolumab plus ipilimumab (NIVO+IPI) vs sunitinib (SUN) for first-line treatment of advanced renal cell carcinoma (aRCC): Long-term follow-up data from the phase 3 CheckMate 214 trial. Journal of Clinical Oncology. 2024;42:363-.

[13] Freedland SJ, Luz MdA, Giorgi UD, Gleave M, Gotto GT, Pieczonka CM, et al. Improved Outcomes with Enzalutamide in Biochemically Recurrent Prostate Cancer. New England Journal of Medicine. 2023;389:1453-65.

[14] Kyriakopoulos CE, Chen YH, Carducci MA, Liu G, Jarrard DF, Hahn NM, et al. Chemohormonal Therapy in Metastatic Hormone-Sensitive Prostate Cancer: Long-Term Survival Analysis of the Randomized Phase III E3805 CHAARTED Trial. J Clin Oncol. 2018;36:1080-7.

[15] Fizazi K, Tran N, Fein L, Matsubara N, Rodriguez-Antolin A, Alekseev BY, et al. Abiraterone plus Prednisone in Metastatic, Castration-Sensitive Prostate Cancer. New England Journal of Medicine. 2017;377:352-60.

[16] Smith MR, Hussain M, Saad F, Fizazi K, Sternberg CN, Crawford ED, et al. Darolutamide and Survival in Metastatic, Hormone-Sensitive Prostate Cancer. New England Journal of Medicine. 2022;386:1132-42.

[17] Tannock IF, Wit Rd, Berry WR, Horti J, Pluzanska A, Chi KN, et al. Docetaxel plus Prednisone or Mitoxantrone plus Prednisone for Advanced Prostate Cancer. New England Journal of Medicine. 2004;351:1502-12.

[18] Fizazi K, Scher HI, Molina A, Logothetis CJ, Chi KN, Jones RJ, et al. Abiraterone acetate for treatment of metastatic castration-resistant prostate cancer: final overall survival analysis of the COU-AA-301 randomised, double-blind, placebo-controlled phase 3 study. Lancet Oncol. 2012;13:983-92.

[19] Scher HI, Fizazi K, Saad F, Taplin M-E, Sternberg CN, Miller K, et al. Increased Survival with Enzalutamide in Prostate Cancer after Chemotherapy. New England Journal of Medicine. 2012;367:1187-97.

[20] Mateo J, Bono JSd, Fizazi K, Saad F, Shore N, Sandhu S, et al. Olaparib for the Treatment of Patients With Metastatic Castration-Resistant Prostate Cancer and Alterations in BRCA1 and/or BRCA2 in the PROfound Trial. Journal of Clinical Oncology. 2024;42:571-83.

[21] Bono Jd, Mateo J, Fizazi K, Saad F, Shore N, Sandhu S, et al. Olaparib for Metastatic Castration-Resistant Prostate Cancer. New England Journal of Medicine. 2020;382:2091-102.

[22] Saad F, Clarke NW, Oya M, Shore N, Procopio G, Guedes JD, et al. Olaparib plus abiraterone versus placebo plus abiraterone in metastatic castration-resistant prostate cancer (PROpel): final prespecified overall survival results of a randomised, double-blind, phase 3 trial. Lancet Oncol. 2023;24:1094-108.

[23] Kitamura H, Hinotsu S, Tsukamoto T, Shibata T, Mizusawa J, Kobayashi T, et al. Effect of neoadjuvant chemotherapy on health-related quality of life in patients with muscle-invasive bladder cancer: results from JCOG0209, a randomized phase III study. Jpn J Clin Oncol. 2020;50:1464-9.

[24] Huddart RA, Hall E, Lewis R, Porta N, Crundwell M, Jenkins PJ, et al. Patient-reported Quality of Life Outcomes in Patients Treated for Muscle-invasive Bladder Cancer with Radiotherapy ± Chemotherapy in the BC2001 Phase III Randomised Controlled Trial. Eur Urol. 2020;77:260-8.

[25] Gupta S, Loriot Y, Heijden MSVD, Bedke J, Valderrama BP, Kikuchi E, et al. Patient-reported outcomes (PROs) from a randomized, phase 3 trial of enfortumab vedotin plus pembrolizumab (EV+P) versus platinum-based chemotherapy (PBC) in previously untreated locally advanced or metastatic urothelial cancer (la/mUC). Journal of Clinical Oncology. 2024;42:4502-.

[26] Choueiri TK, Tomczak P, Park SH, Venugopal B, Symeonides S, Hajek J, et al. Patient-Reported Outcomes in KEYNOTE-564: Adjuvant Pembrolizumab Versus Placebo for Renal Cell Carcinoma. Oncologist. 2024;29:142-50.

[27] Motzer RJ, Hutson TE, Tomczak P, Michaelson MD, Bukowski RM, Rixe O, et al. Sunitinib versus Interferon Alfa in Metastatic Renal-Cell Carcinoma. New England Journal of Medicine. 2007;356:115-24.

[28] Castellano D, del Muro XG, Pérez-Gracia JL, González-Larriba JL, Abrio MV, Ruiz MA, et al. Patient-reported outcomes in a phase III, randomized study of sunitinib versus interferon-{alpha} as first-line systemic therapy for patients with metastatic renal cell carcinoma in a European population. Ann Oncol. 2009;20:1803-12.

[29] Motzer R, Porta C, Alekseev B, Rha SY, Choueiri TK, Mendez-Vidal MJ, et al. Health-related quality-of-life outcomes in patients with advanced renal cell carcinoma treated with lenvatinib plus pembrolizumab or everolimus versus sunitinib (CLEAR): a randomised, phase 3 study. Lancet Oncol. 2022;23:768-80.

[30] Bedke J, Rini BI, Plimack ER, Stus V, Gafanov R, Waddell T, et al. Health-related Quality of Life Analysis from KEYNOTE-426: Pembrolizumab plus Axitinib Versus Sunitinib for Advanced Renal Cell Carcinoma. Eur Urol. 2022;82:427-39.

[31] Cella D, Motzer RJ, Suarez C, Blum SI, Ejzykowicz F, Hamilton M, et al. Patient-reported outcomes with first-line nivolumab plus cabozantinib versus sunitinib in patients with advanced renal cell carcinoma treated in CheckMate 9ER: an open-label, randomised, phase 3 trial. Lancet Oncol. 2022;23:292-303.

[32] Cella D, Grünwald V, Escudier B, Hammers HJ, George S, Nathan P, et al. Patient-reported outcomes of patients with advanced renal cell carcinoma treated with nivolumab plus ipilimumab versus sunitinib (CheckMate 214): a randomised, phase 3 trial. Lancet Oncol. 2019;20:297-310.

[33] Freedland Stephen J, Gleave M, De Giorgi U, Rannikko A, Pieczonka Christopher M, Tutrone Ronald F, et al. Enzalutamide and Quality of Life in Biochemically Recurrent Prostate Cancer. NEJM Evidence. 2023;2:EVIDoa2300251.

[34] Sentana-Lledo D, Chu X, Jarrard DF, Carducci MA, DiPaola RS, Wagner LI, et al. Patient-reported Quality of Life and Survival Outcomes in Prostate Cancer: Analysis of the ECOG-ACRIN E3805 Chemohormonal Androgen Ablation Randomized Trial (CHAARTED). Eur Urol Oncol. 2024.

[35] Chi KN, Protheroe A, Rodríguez-Antolín A, Facchini G, Suttman H, Matsubara N, et al. Patient-reported outcomes following abiraterone acetate plus prednisone added to androgen deprivation therapy in patients with newly diagnosed metastatic castration-naive prostate cancer (LATITUDE): an international, randomised phase 3 trial. Lancet Oncol. 2018;19:194-206.

[36] Fizazi K, Smith M, Hussain M, Saad F, Sternberg C, Crawford E, et al. 1360MO Quality of life and patient-relevant endpoints with darolutamide in the phase III ARASENS study. Annals of Oncology. 2022;33:S1162.

[37] Harland S, Staffurth J, Molina A, Hao Y, Gagnon DD, Sternberg CN, et al. Effect of abiraterone acetate treatment on the quality of life of patients with metastatic castration-resistant prostate cancer after failure of docetaxel chemotherapy. Eur J Cancer. 2013;49:3648-57.

[38] Cella D, Ivanescu C, Holmstrom S, Bui CN, Spalding J, Fizazi K. Impact of enzalutamide on quality of life in men with metastatic castration-resistant prostate cancer after chemotherapy: additional analyses from the AFFIRM randomized clinical trial. Ann Oncol. 2015;26:179-85.

[39] Thiery-Vuillemin A, de Bono J, Hussain M, Roubaud G, Procopio G, Shore N, et al. Pain and health-related quality of life with olaparib versus physician's choice of next-generation hormonal drug in patients with metastatic castration-resistant prostate cancer with homologous recombination repair gene alterations (PROfound): an open-label, randomised, phase 3 trial. The Lancet Oncology. 2022;23:393-405.

[40] Thiery-Vuillemin A, Saad F, Armstrong AJ, Oya M, Vianna K, Özgüroğlu M, et al. Health-related quality of life (HRQoL) and pain outcomes for patients (pts) with metastatic castration-resistant prostate cancer (mCRPC) who received abiraterone (abi) and olaparib (ola) versus (vs) abi and placebo (pbo) in the phase III PROpel trial. Journal of Clinical Oncology. 2023;41:5012-.

[41] Saad F, Thiery-Vuillemin A, Wiechno P, Alekseev B, Sala N, Jones R, et al. Patient-reported outcomes with olaparib plus abiraterone versus placebo plus abiraterone for metastatic castration-resistant prostate cancer: a randomised, double-blind, phase 2 trial. The Lancet Oncology. 2022;23:1297-307.
